# Novel strains of typical enteropathogenic Escherichia coli (tEPEC) in Pteropus bats, including the first report of a phylogroup G tEPEC

**DOI:** 10.1099/mgen.0.001469

**Published:** 2025-08-11

**Authors:** Fiona K. McDougall, Michelle L. Power

**Affiliations:** 1 Macquarie University, Sydney, NSW, School of Natural Sciences, Faculty of Science and Engineering, Australia

**Keywords:** *bfpA*, *bfp* operon, flying fox, fruit bat, one health

## Abstract

Typical enteropathogenic *Escherichia coli* (tEPEC) is an important cause of prolonged diarrhoea in human infants. Humans are considered the primary reservoir and the only natural host of human-associated tEPEC. Recently, bat-specific tEPEC lineages were consistently detected in an Australian fruit bat species (*Pteropus poliocephalus*; grey-headed flying fox), indicating that *P. poliocephalus* is also a natural tEPEC host. tEPEC are differentiated from other pathogenic *E. coli* by the presence of the bundle-forming pilus (*bfp*) operon. In this study, we characterize two additional bat-associated tEPEC strains, the first (FF593) from *P. poliocephalus* and the second (FF618) from another Australian *Pteropus* species, *Pteropus conspicillatus* (spectacled flying fox). Whole-genome sequencing of the two bat tEPEC isolates revealed that both strains belonged to novel bat tEPEC lineages (FF593 to Clade F and FF618 to Clade E) and both carried novel *bfp* operons. FF593 is the first report of a phylogroup G tEPEC strain, and FF618 was phylogenetically related to a human aEPEC lineage (ST1041). Phylogenetic analysis of 11 bat and 11 human *bfp* operons determined that bat and human variants belong to separate lineages but share an evolutionary origin. This study provides further evidence that diverse bat-specific tEPEC lineages and *bfp* operons have evolved in Australian *Pteropus* bats and suggests that multiple species of Australian *Pteropus* bats are natural hosts for bat-specific tEPEC strains.

**Impact Statement**

Humans have traditionally been considered the only natural host of typical enteropathogenic *Escherichia coli* (tEPEC), an important cause of diarrhoea in human infants. Recently, tEPEC were consistently detected in an Australian fruit bat species (the grey-headed flying fox), suggesting that they are also natural tEPEC hosts. This study provided further evidence that tEPEC in Australian fruit bats and tEPEC in humans have evolved independently, but they share common evolutionary origins. We also describe the first report of a novel tEPEC lineage, which belongs to *E. coli* phylogroup G. One tEPEC strain was detected in a second species of Australian fruit bat (the spectacled flying fox), suggesting that multiple species of Australian fruit bats are natural hosts of bat-specific tEPEC.

## Data Summary

Full-length *bfp* operon sequences carried by tEPEC isolates FF593 and FF618 have been uploaded to GenBank (accession numbers PV285313 and PV285312, respectively). Raw sequence reads for both tEPEC isolates have been uploaded to the NCBI Sequence Read Archive (SRA) under BioProject ID PRJNA613581 (BioSample accessions SAMN47229884 and SAMN47229885) (available at https://www.ncbi.nlm.nih.gov/bioproject/?term=PRJNA613581), and assembled sequences are available in EnteroBase under Barcodes ESC_HA7318AA and ESC_HA7322AA (https://enterobase.warwick.ac.uk/species/index/ecoli) (Table S1, available in the online Supplementary Material).

## Introduction

Typical enteropathogenic *Escherichia coli* (tEPEC) is an important cause of prolonged diarrhoea in human infants and is associated with infant mortality in low- and middle-income countries [1,2]. tEPEC are defined by two key virulence factors: a locus for enterocyte effacement (LEE) pathogenicity island and the EPEC adherence factor plasmid (pEAF) [3]. The presence of pEAF differentiates tEPEC from atypical enteropathogenic *E. coli* (aEPEC), which only carry LEE [3]. LEE is a ~35 kb chromosomal locus which encodes 41 genes including the intimin encoding *eae* gene and other genes encoding an outer membrane protein, a type 3 secretion system and several type 3 secreted effectors [4]. LEE has been independently acquired by diverse *E. coli* lineages, resulting in the evolution of distinct EPEC lineages which have specific LEE chromosomal insertion sites, LEE variants and *eae* allele types [4,6]. The pEAF plasmid encodes the bundle-forming pilus (*bfp*) operon (encodes 14 genes including *bfpA*) and plasmid-encoded regulators (*per*) [7,8]. As with LEE, multiple variants of pEAF and *bfpA* alleles have also evolved; however, pEAF and *bfpA* are not highly conserved within tEPEC lineages [6].

The majority of human-associated tEPEC belong to well-described classical serogroups [3], which predominantly cluster in three phylogenetic lineages designated EPEC1, EPEC2 and EPEC4 [9]. Humans are considered to be the primary reservoir and the only natural host of these human-associated tEPEC lineages [10]. However, a recent study consistently detected tEPEC in an Australian fruit bat species (*Pteropus poliocephalus*) over a 4-year time period and estimated tEPEC prevalence at 18.0%, suggesting that wild *P. poliocephalus* are also a natural tEPEC host [11]. Phylogenetic analysis of 13 tEPEC from wild *P. poliocephalus* showed that the bat tEPEC shared evolutionary origins and *eae* allele types with human EPEC [11] but did not belong to the classical serogroups associated with human tEPEC [3]. The bat tEPEC predominantly belonged to novel tEPEC lineages (designated Clades A, C and D) and typically harboured novel sequence types (STs) and novel *bfpA* variants, suggesting that bat tEPEC have evolved independently [11].

Here, we extend knowledge of bat-specific tEPEC lineages through the whole-genome sequencing (WGS) of two additional tEPEC isolates from *Pteropus* bats housed in wildlife rehabilitation facilities.

## Methods

### Bat tEPEC isolates

Old World fruit bats and flying foxes belong to the family Pteropodidae (Yinpterochiroptera) [12] which comprises 45 genera and 202 species. The genus *Pteropus* is the largest of the Pteropodidae family with 64 species [13], of which 4 species occur on mainland Australia [14].

Two tEPEC isolates (FF593 and FF618) from an existing bat *E. coli* collection were used in this study. FF593 and FF618 were identified as tEPEC using a duplex PCR (targeting the *eae* and *bfpA* genes) as previously described [11]. The tEPEC isolates were cultured from the faeces of two Australian *Pteropus* bat species: FF593 from *P. poliocephalus* [grey-headed flying fox (GHFF)] and FF618 from *Pteropus conspicillatus* [spectacled flying fox (SFF)]. The two individual bats were sick or injured and undergoing care at bat rehabilitation facilities (GHFF at Wambina Flying Fox Sanctuary, New South Wales and SFF at Tolga Bat Hospital, Queensland).

### WGS of tEPEC isolates

Genomic DNA was extracted from the two tEPEC isolates using the ISOLATE II Genomic DNA Kit (Bioline, London, UK) and sequenced using the Illumina MiSeq platform, as previously described [11]. Raw sequence reads were assembled using CLC Genomics Workbench 8 (CLC Bio, Aarhus, Denmark), and assembled DNA sequences were analysed for virulence factors using VirulenceFinder 2.0 [15] (available at http:// www.genomicepidemiology.org/services/) and Abricate VFDB [16] available on the Galaxy Australia platform (https://usegalaxy.org.au/) and antimicrobial resistance genes using ResFinder 4 [17,18] (available at https://genepi.dk) and assigned an ST using the Achtman 7 Gene Multilocus Sequence Type (MLST) scheme and an O:H serotype using the Serotype Prediction tools, both available in EnteroBase (http://enterobase.warwick.ac.uk/species/index/ecoli) [19]. Full-length *bfp* operon and LEE sequences were extracted using Geneious Prime (Biomatters Limited, Auckland, New Zealand), as previously described [11]. Full-length *eae* and *bfpA* gene sequences were extracted from LEE and *bfp* operon sequences, respectively, using Geneious Prime (Biomatters Limited), and allele types were determined by performing blastn searches (available at https://blast.ncbi.nlm.nih.gov/Blast.cgi) against a panel of *eae* and *bfpA* reference alleles (Tables S2 and S3, respectively). Phylogroup was determined using the ClermonTyping tool in EnteroBase [20] (available at https://enterobase.warwick.ac.uk/species/index/ecoli) [19].

### Phylogenetic analysis of tEPEC strains FF593 and FF618

Phylogenetic relationships between tEPEC isolates FF593 and FF618 and 13 previously characterized tEPEC isolates from wild GHFF (NCBI SRA BioProject ID PRJNA613581) were inferred using a maximum likelihood based on RAxML (version 8) [21] of non-repetitive core SNPs (minimum presence 95%) using the EnteroBase SNP Project dendrogram module against a reference genome (*E. coli* LF82 chromosome, complete sequence 4,773,108 bp, EnteroBase Barcode ESC_CA9027AA) [19]. The tEPEC phylogenetic tree was annotated using Interactive Tree of Life (iTOL) v6(https://itol.embl.de/) [22]. Individual sample SRA and EnteroBase Barcodes for all 15 bat tEPEC isolates are available in Table S1.

Isolates phylogenetically related to tEPEC strains FF593 (ST7791) and FF618 (ST1041) were identified in EnteroBase by searching for STs ST7791 (with up to three allele mismatches) and ST1041, respectively, using the Achtman seven-gene MLST scheme query tool (available at https://enterobase.warwick.ac.uk/species/index/ecoli) [19]. Phylogenetic relationships between bat tEPEC (FF593 and FF618) and respective related isolates identified in EnteroBase were performed using GrapeTree to construct rapid neighbour joining (RapidNJ) minimum spanning trees based on the core genome multilocus sequence typing (cgMLST) V1+Hierarchical Clustering (HierCC) V1 scheme (available at https://enterobase.warwick.ac.uk/species/index/ecoli) [23].

Assembled genomes of EPEC isolates related to FF593 and FF618 were downloaded from EnteroBase (https://enterobase.warwick.ac.uk/species/index/ecoli) or GenBank (https://www.ncbi.nlm.nih.gov/biosample/?term=) and imported into Geneious Prime (Biomatters Limited). Isolate *eae* and *bfpA* allele types were then determined as for FF593 and FF618 (described above).

### Phylogenetic analysis of bat *bfp* operons

To identify *bfp* operons that were closely related to bat-associated *bfp* operons, blastn searches (available at https://blast.ncbi.nlm.nih.gov/Blast.cgi) of bat *bfp* operon sequences (GenBank accessions PV28531, PV285313 and OM925979 to OM925987) were performed. All *bfp* operons from *E. coli* or *Escherichia albertii* with 100% coverage and >90% identity to bat-associated *bfp* operons were downloaded from GenBank (*n*=14; accessions AB024946, AP014804, CP021211, CP042948, CP059841, CP117640, CP117645, CP117660, CP148747, CP164309, DQ388534, ECU27184, FM180569 and Z68186). To determine the phylogenetic relationships between bat-, human- and bird-associated *bfp* operons, a phylogenetic tree was constructed from all *bfp* operon sequences using RAxML (version 8) [21] tree builder in Geneious Prime 2023 with nt model GTR CAT and rapid hill-climbing algorithm (Biomatters Limited).

## Results

### Characterization of tEPEC isolates FF593 and FF618

**Fig. 1.**
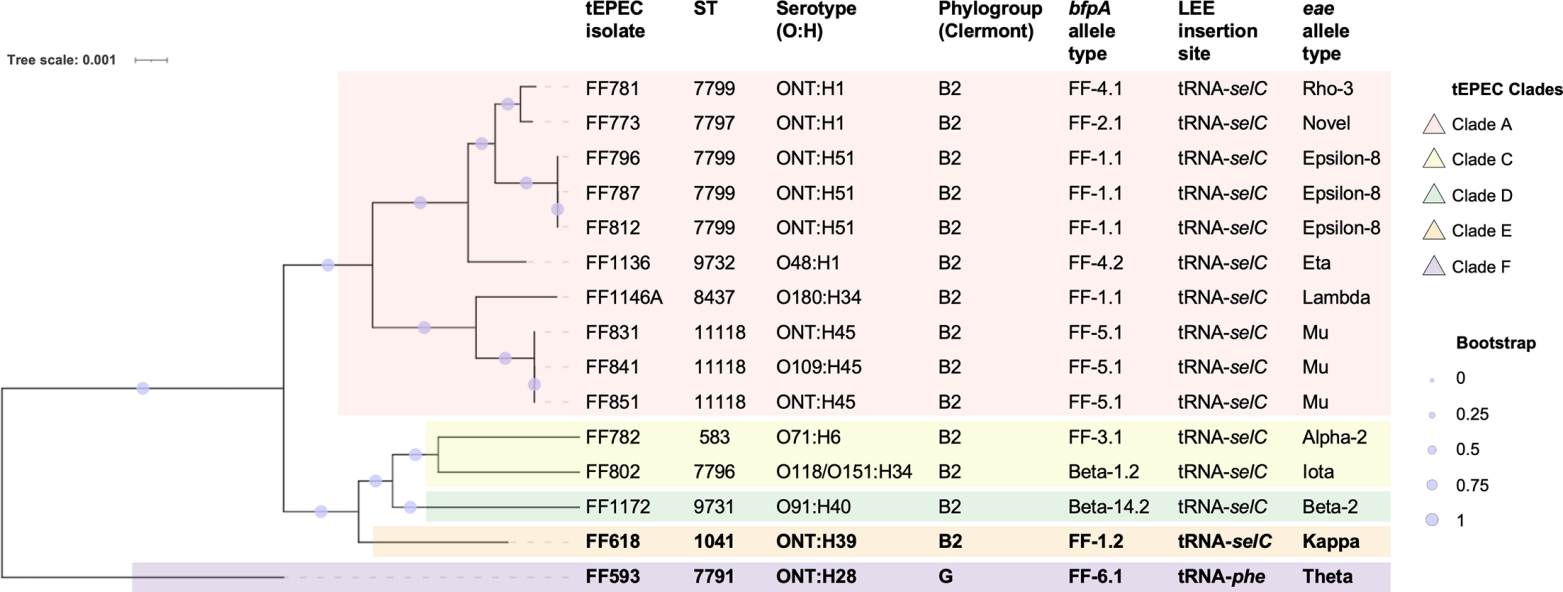
Phylogeny of the two tEPEC strains from *Pteropus* bats in care (FF593 from an adult GHFF and FF618 from an adult SFF) and 13 tEPEC strains from wild GHFFs, showing that *Pteropus*-associated tEPEC strains belong to 5 different clades. The two isolates from this study (FF593 and FF618) are highlighted in bold font. The maximum-likelihood tree was based on RAxML (version 8) of non-repetitive core SNPs using the EnteroBase SNP Project dendrogram module against a reference genome (EnteroBase Barcode ESC_CA9027AA) which has been removed from the SNP tree image for clarity. Scale bar indicates the number of substitutions per site.

The bat tEPEC isolate FF593 was assigned as ST7791 serotype ONT:H28, and tEPEC FF618 was assigned as ST1041 serotype ONT:H39. FF618 belonged to phylogroup B2, whereas tEPEC FF593 belonged to phylogroup G (Fig. 1).

The constructed SNP phylogeny tree placed both FF593 and FF618 in two distinct lineages from the 13 previously identified tEPEC from wild GHFF (Fig. 1). Neither FF593 nor FF618 was closely related to any of the 13 previously identified tEPEC, which belonged to 3 different tEPEC lineages designated Clade A, Clade C and Clade D (Fig. 1). FF618 belonged to a tEPEC lineage designated Clade E, which was most closely related to Clades C and D, and FF593 belonged to a divergent lineage designated Clade F (Fig. 1).

Both bat tEPEC isolates (FF593 and FF618) harboured novel *bfpA* alleles. FF618 carried a *bfpA* allele, designated FF-1.2, which differed by one SNP to the *bfpA* FF-1.1 allele previously identified in Clade A tEPEC from wild GHFF (GenBank accessions OM925981 and OM925982) (Fig. 1). FF618 carried a novel *bfpA* allele designated FF-6.1 (Fig. 1), which most closely matched (93.7%) the bat FF-3.1 *bfpA* reference allele (GenBank accession OM925984).

FF593 carried an *eae* allele type which had 99.9% identity to the theta *eae* reference allele (GenBank accession AB334563) and FF618 carried an *eae* allele type which had 99.9% identity to the kappa *eae* reference allele (GenBank accession AJ308552) (Fig. 1). The LEE insertion site for FF618 was determined to be tRNA-selC (as for all 13 previously identified tEPEC from wild GHFF), whereas the FF593 LEE insertion site was tRNA-phe (Fig. 1).

In addition to the *bfp* operon (encodes 14 genes including *bfpA*) and LEE encoded genes, which include *eae* (intimin), *esc* genes (type III secretion system), *esp* genes (effectors) and *tir* (translocated intimin receptor), FF593 harboured *paa* (porcine attaching and effacing-associated) gene and both tEPEC isolates harboured multiple non-LEE encoded effectors (FF593 carried *nleF* and *nleH1*; FF618 carried *nleH1* and *cif*). FF593 carried additional virulence genes including *astA* and *usp* (toxins)*, ibeA* (invasin) and both tEPEC isolates carried *traT* (protectin). Neither isolate carried any acquired antimicrobial resistance genes nor point mutations conferring antimicrobial resistance.

### Phylogeny of bat tEPEC (FF593 and FF618) and related *E. coli* strains

**Fig. 2.**
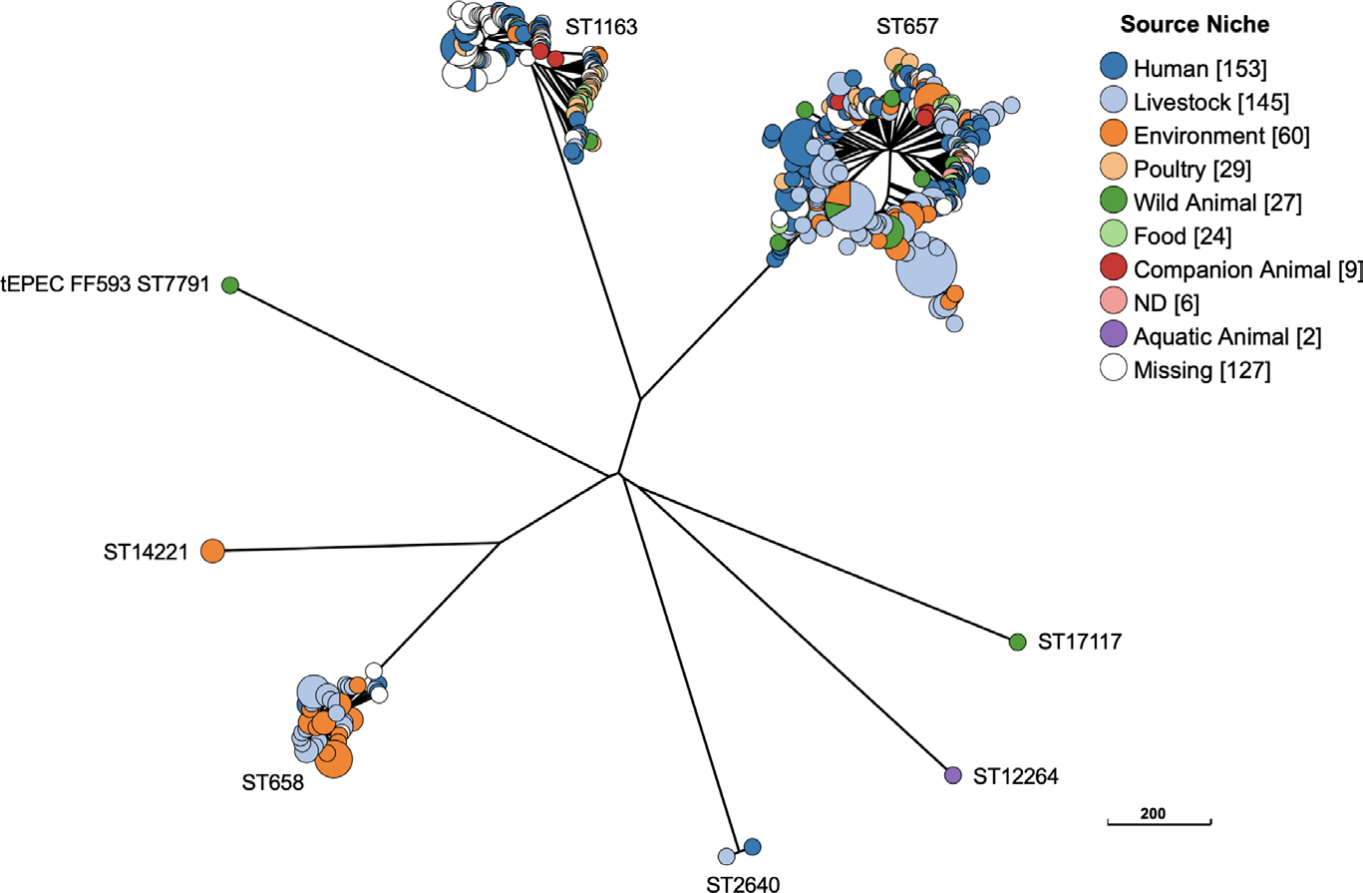
Clade F (phylogroup G) GrapeTree. Phylogenetic analysis of bat tEPEC isolate FF593 (ST7791) and closely related phylogroup G *E. coli* isolates identified in EnteroBase (*n*=582 isolates). *E. coli* ST of each phylogroup G lineage is indicated adjacent to the respective clusters. GrapeTree phylogeny was constructed using a RapidNJ minimum spanning tree based on the cgMLST V1+Hierarchical Clustering (HierCC) V1 scheme. Scale bar indicates the number of cgMLST allelic differences.

FF593 was identified as the sole ST7791 isolate in EnteroBase; however, a search for closely related isolates with up to three allele mismatches (Achtman 7 Gene MLST scheme) determined ST7791 had two or three allele mismatches with ST658, ST657 and ST1163 (all phylogroup G). The Clade F GrapeTree phylogeny of FF593 and closely related phylogroup G isolates identified in EnteroBase (*n*=582) placed FF593 in a divergent lineage within a large clade (Fig. 2). Among these closely related phylogroup G isolates (*n*=582), FF593 was determined to be the only EPEC isolate. A further search of all phylogroup G *E. coli* isolates in EnteroBase (*n*=5,730) identified two aEPEC strains (ST117 Barcode ESC_TA6906AA and ST189 Barcode ESC_OA7818AA); however, both carried different *eae* allele types (alpha-1 and beta-1, respectively) in comparison to FF593 (theta). One further phylogroup G aEPEC isolate (ST2712) was identified in GenBank (BioSample: SAMEA6083517); however, despite also being sourced in Australia and having the same serotype (ONT:H28), ST2712 also carried a different *eae* allele type (beta-2) to FF593 (theta).

**Fig. 3.**
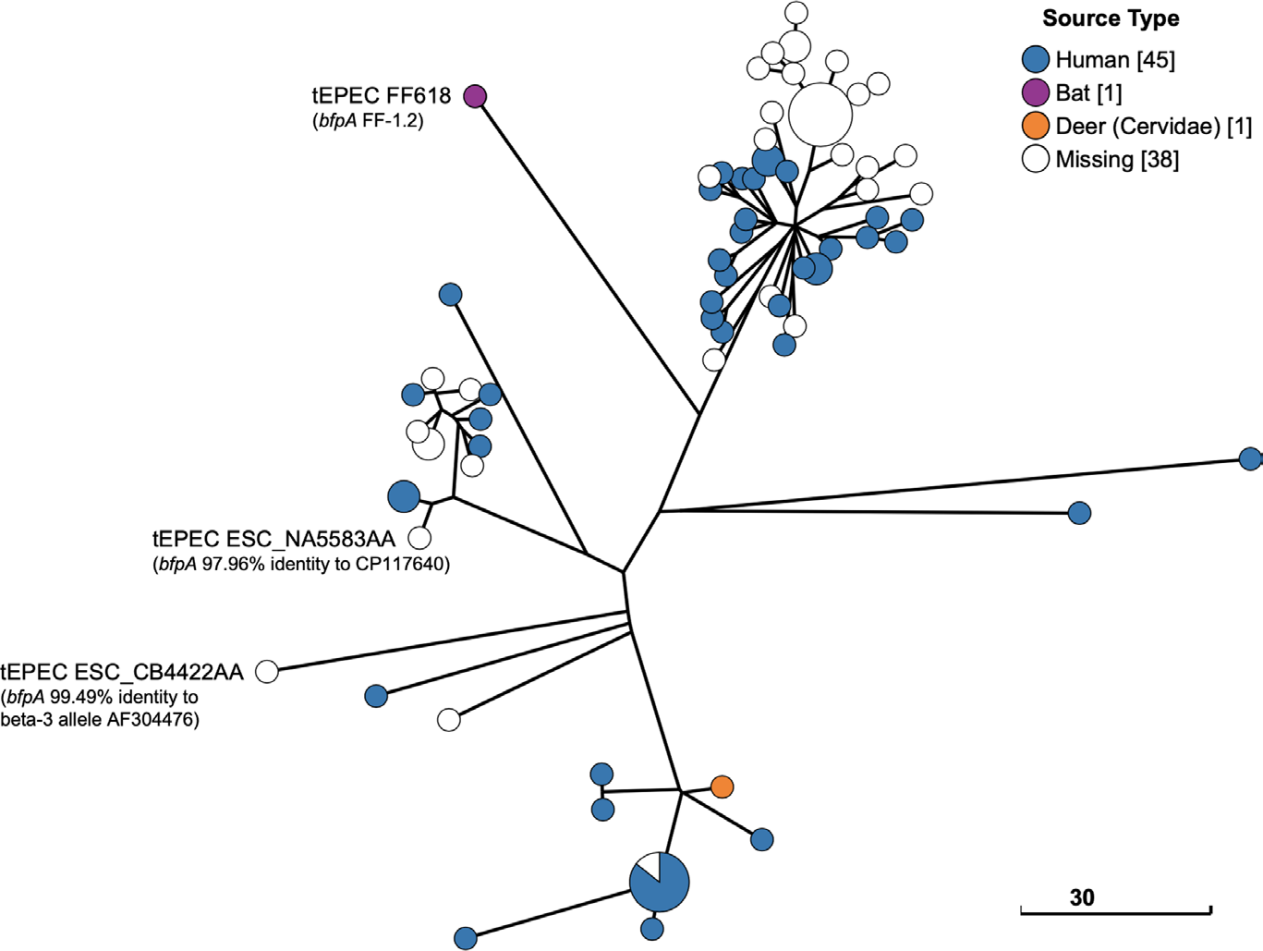
Clade E (phylogroup B2) GrapeTree. Phylogenetic analysis of bat tEPEC isolate FF618 (ST1041) and 84 closely related isolates identified in EnteroBase. All 85 ST1041 isolates, including FF618, were EPEC (tEPEC *n*=*3* and aEPEC *n*=82), all carried the kappa *eae* allele and the predominant serotype was O157:H39. The positions of FF618 and two additional ST1041 tEPEC isolates (EnteroBase Barcodes ESC_NA5583AA and ESC_CB4422AA) are indicated. All three tEPEC carried different *bfpA* allele types (as indicated in tEPEC isolate labels). GrapeTree phylogeny was constructed using a RapidNJ minimum spanning tree based on the cgMLST V1+Hierarchical Clustering (HierCC) V1 scheme. Scale bar indicates the number of cgMLST allelic differences.

FF618 (ST1041 ONT:H39) was one of three ST1041 tEPEC identified in EnteroBase; however, the other two tEPEC (EnteroBase Barcodes ESC_NA5583AA and ESC_CB4422AA) carried different *bfpA* allele types in comparison to FF618. blastn searches determined that ESC_NA5583AA carried a *bfpA* variant which showed 97.96% identity to an undefined *bfpA* allele from *E. albertii* CP117640 and ESC_CB4422AA carried a *bfpA* variant which had the closest match (99.49%) to beta-3 allele AF304476, whereas FF618 carried a FF-1.2 *bfpA* allele. A further 82 *E. coli* ST1041 isolate assemblies were retrieved from EnteroBase, of which all were aEPEC. All 85 ST1041 aEPEC and tEPEC, including FF618, carried the kappa *eae* allele type and the predominant serotype was O157:H39. The Clade E GrapeTree phylogeny of FF618 and the 84 ST1041 isolates from EnteroBase placed FF618 in a divergent lineage, with the two additional tEPEC (ESC_NA5583AA and ESC_CB4422AA) also belonging to different lineages (Fig. 3).

### Phylogeny of bat *bfp* operons

blastn searches of the full-length *bfp* operon sequences for FF593 and FF618 (GenBank accessions PV285313 and PV285312, respectively) determined that both tEPEC carried novel *bfp* operon variants. The FF618 *bfp* operon (designated variant FF-1.3) differed by one and two SNPs to two previously identified bat *bfp* operons, variant FF-1.2 (GenBank accession OM925982) and variant FF-1.1 (GenBank accession OM925981), whereas the FF593 *bfp* operon (designated variant FF-6.1) was most closely related (98.38% identity) to the bat *bfp* operon variant FF-2.1 (GenBank accession OM925983).

**Fig. 4.**
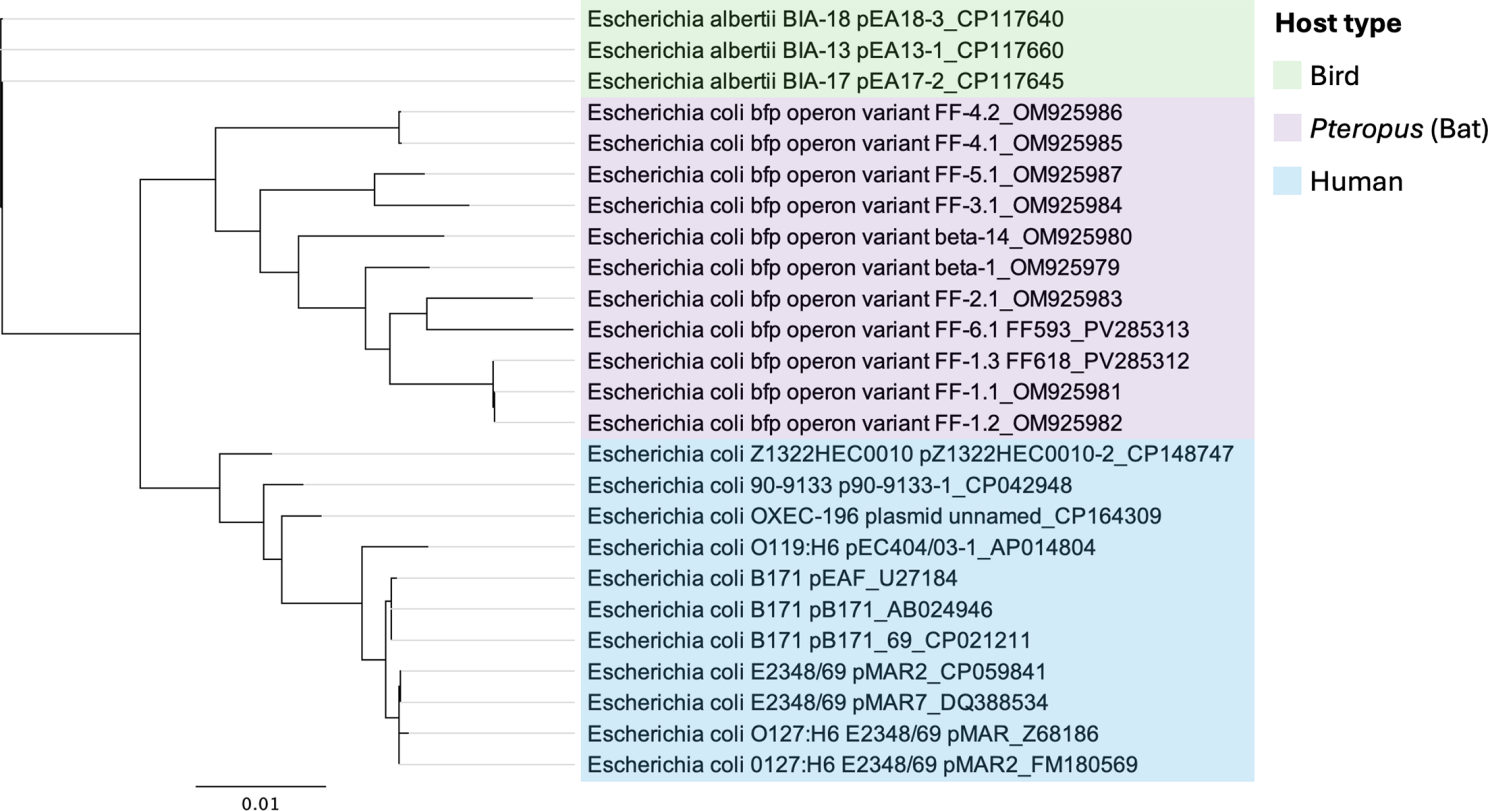
Phylogenetic tree depicting the relationships between bat-, human- and bird-associated *bfp* operons. The tree is comprised of 11 *Pteropus* (bat) *bfp* operons originating from Australia and 14 closely related *bfp* operons from internationally sourced human-associated *E. coli* (*n*=11) and bird (*G. grus*; common crane) sourced *E. albertii* (*n*=3) originating from Poland. All 14 non-bat *bfp* operons were obtained from GenBank blastn searches, and only *bfp* operons from *E. coli* or *E. albertii* with 100% coverage and >90% identity to bat-associated *bfp* operons were included in the phylogenetic tree. GenBank accessions for each *bfp* operon sequence are provided in the individual tree tip labels. The phylogenetic tree was constructed using RAxML tree builder with nt model GTR CAT and rapid hill-climbing algorithm. Scale bar indicates the number of substitutions per site.

The inferred phylogeny of full-length *bfp* operon sequences from *Pteropus* (bat) tEPEC (*n*=11), human *E. coli* (*n*=11) and bird (*Grus grus*; common crane) *E. albertii* (*n*=3) showed that the bat, human and bird *bfp* operons belonged to three different lineages; however, the bat and human *bfp* operon variants did share a common evolutionary origin (Fig. 4).

## Discussion

This study revealed two additional bat-specific tEPEC strains, with one detected in a new *Pteropus* bat species (*P. conspicillatus*). The bat tEPEC isolates included one novel ST, and both carried novel *bfpA* allele types, which is consistent with the characteristics of tEPEC previously detected in wild *P. poliocephalus* in Australia [11].

Phylogenetic analysis has determined that bat tEPEC strains belong to five clades, designated Clade A, Clade C, Clade D [11], Clade E and Clade F (this study). Notably, the tEPEC strain in Clade F (FF593 ST7791 ONT:H28) belonged to phylogroup G, whereas all other bat tEPEC belonged to phylogroup B2 [11]. Phylogroup G is closely related to phylogroup B2, and specific lineages are characterized by carriage of extra-intestinal virulence genes or intestinal virulence genes, including Shiga-toxins (*stx2*) but rarely *eae* [24]. The bat strain FF593 (ST7791 ONT:H28) appears to be the first report of a phylogroup G tEPEC strain, which also carries a novel *bfpA* allele type (FF-6.1).

Phylogenetic analysis and *eae* allele typing showed that the Clade E tEPEC strain (FF618 ST1041) has a shared evolutionary lineage with human-associated aEPEC (ST1041), rather than with a human-associated tEPEC lineage, which is consistent with the phylogenies of previously identified bat tEPEC in Clades A, C and D [11]. FF618 carries a bat-specific *bfp* operon (FF-1.3) which is almost identical to *bfp* operons previously identified in multiple strains of Clade A tEPEC detected in wild *P. poliocephalus* [11]. This finding provides further evidence for transmission of bat-specific *bfp* operons (and presumably pEAF plasmids) between diverse bat EPEC strains. Additionally, the tEPEC FF618 strain was detected in *P. conspicillatus*, whose geographical range very rarely overlaps with *P. poliocephalus* [14,25], suggesting that bat-specific tEPEC and *bfp* operons have been circulating within Australian *Pteropus* populations for a long time.

The inferred *bfp* operon phylogeny determined that bat and human *bfp* operon variants share an evolutionary origin but belong to separate lineages, indicating that bat-specific *bfp* operons have evolved independently to human *bfp* operons. This distinct bat-specific *bfp* operon lineage further supports that *Pteropus* bats have evolved bat-specific tEPEC strains.

The 13 tEPEC previously identified in wild *P. poliocephalus* exhibited localized adherence to human epithelial cells (HEp-2 cells) *in vitro*, a characteristic associated with tEPEC pathology [10], suggesting bat tEPEC have zoonotic potential and may pose a public health risk [11]. Although the two bat tEPEC identified in this study did not undergo HEp-2 *in vitro* assays, it would be prudent to also consider these strains as potential zoonotic pathogens. However, to date, no bat-specific tEPEC strains or bat-specific *bfpA* alleles have been reported in studies which characterized human-associated tEPEC isolates.

In conclusion, this study further demonstrates that diverse bat-specific tEPEC lineages and *bfp* operons have evolved in Australian *Pteropus* bats and indicates that multiple species of Australian *Pteropus* bats are natural hosts for bat-specific tEPEC strains. Additional research is warranted to investigate the prevalence and diversity of tEPEC in all Australian *Pteropus* bat species.

## Supplementary material

10.1099/mgen.0.001469 Uncited Supplementary Material 1.

## Abbreviations

aEPEC: atypical enteropathogenic *Escherichia coli*
*bfp*: bundle-forming pilus
cgMLST: core genome multilocus sequence typing
GHFF: grey-headed flying fox
MLST: Multilocus Sequence Type
RapidNJ: rapid neighbour joining
SFF: spectacled flying fox
ST: sequence type
tEPEC: typical enteropathogenic *Escherichia coli*
WGS: whole-genome sequencing
