# Supplementary material for: Novel strains of typical enteropathogenic Escherichia coli (tEPEC) in Pteropus bats, including the first report of a phylogroup G tEPEC
Source: Microb Genom. 2025 Aug 11;11(8):001469. doi: 10.1099/mgen.0.001469 (PMC12452172; doi:10.1099/mgen.0.001469)
Supplement: Uncited Supplementary Material 1. [file mgen-11-01469-s001.pdf]

Supplementary Tables

Title:

Novel strains of typical enteropathogenic *Escherichia coli* (tEPEC) in *Pteropus* bats, including the first report of a phylogroup G tEPEC

Author names

Fiona K McDougall[1] and Michelle L Power[1]

Author affiliations

[1] School of Natural Sciences, Faculty of Science and Engineering, Macquarie University, Sydney, NSW 2109, Australia

Table S1. Individual tEPEC isolate NCBI SRA BioProject and Biosample IDs, and EnteroBase Barcodes for 13 bat tEPEC.

Isolate_ID	ST	Serotype	*bfpA* allele type	Phylogroup	tEPEC clade	Bioproject_ID	Biosample ID	Enterobase Barcode	Reference
FF593	7791	ONT:H28	FF-6.1	G	F	PRJNA613581	SAMN47229884	ESC_HA7318AA	This study
FF618	1041	ONT:H39	FF-1.2	B2	E	PRJNA613581	SAMN47229885	ESC_HA7322AA	This study
FF773	7797	ONT:H1	FF-2.1	B2	A	PRJNA613581	SAMN14409778	ESC_HA7323AA	(1)
FF781	7799	ONT:H1	FF-4.1	B2	A	PRJNA613581	SAMN14409779	ESC_HA7324AA	(1)
FF782	583	O71:H6	FF-3.1	B2	C	PRJNA613581	SAMN14409780	ESC_RA4667AA	(1)
FF787	7799	ONT:H51	FF-1.1	B2	A	PRJNA613581	SAMN14409781	ESC_HA7325AA	(1)
FF796	7799	ONT:H51	FF-1.1	B2	A	PRJNA613581	SAMN14409784	ESC_HA7328AA	(1)
FF802	7796	O118/O151:H34	beta-1.2	B2	C	PRJNA613581	SAMN14409785	ESC_HA7329AA	(1)
FF812	7799	ONT:H51	FF-1.1	B2	A	PRJNA613581	SAMN14409786	ESC_HA7330AA	(1)
FF831	11118	ONT:H45	FF-5.1	B2	A	PRJNA613581	SAMN14409788	ESC_RA4669AA	(1)
FF841	11118	O109:H45	FF-5.1	B2	A	PRJNA613581	SAMN14409789	ESC_RA4670AA	(1)
FF851	11118	ONT:H45	FF-5.1	B2	A	PRJNA613581	SAMN14409790	ESC_RA4668AA	(1)
FF1136	9732	O48:H1	FF-4.2	B2	A	PRJNA613581	SAMN14409807	ESC_JA9913AA	(1)
FF1146A	8437	O180:H34	FF-1.1	B2	A	PRJNA613581	SAMN14409808	ESC_JA9911AA	(1)
FF1172	9731	O91:H40	beta-14.2	B2	D	PRJNA613581	SAMN14409800	ESC_JA9917AA	(1)

Table S2. Reference sequences for *eae* allele typing.

eae allele	GenBank accession	Reference
α (alpha)	AF022236	(1)
α (alpha)	FJ609829	Lacher et al., Unpublished
α2 (alpha-2)	DQ523600	(2)
β (beta)	AJ277443	Benkel and Chakraborty, Unpublished
β2 (beta-2)	DQ523605	(2)
β3 (beta-3)	AJ876654	Blanco et al., Unpublished
β4 (beta-4)	FJ609802	Lacher et al., Unpublished
γ (gamma)	NC002695	(3)
γ (gamma)	DQ523608	(2)
γ2 (gamma-2)	AF025311	(4)
ε (epsilon)	DQ523606	(2)
ε (epsilon)	DQ523612	(2)
ε (epsilon)	AY186750	(5)
ε2 (epsilon-2)	DQ523614	(2)
ε3 (epsilon-3)	FJ609827	Lacher et al., Unpublished
ε4 (epsilon-4)	AJ876651	Blanco et al., Unpublished
ε8 (epsilon-8)	FJ609811	Lacher et al., Unpublished
ζ (zeta)	AJ298279	(6)
ζ (zeta)	AF449417	(7)
ζ2 (zeta-2)	FM872420	(8)
ζ3 (zeta-3)	FM872421	Blanco et al., Unpublished
η (eta)	DQ523604	(2)
η2 (eta-2)	AJ876652	(9)
θ (theta)	AF449418	(7)
θ2 (theta-2)	FM872418	(10)
ι (iota)	DQ523601	(2)
ι (iota)	DQ523602	(2)
ι2 (iota-2)	AY696842	(11)
κ (kappa)	AJ308552	(12)

κ (kappa)	DQ523603	(2)
κ (kappa)	DQ523611	(2)
λ (lambda)	DQ523609	(2)
λ2 (lambda-2)	FJ609808	Lacher et al., Unpublished
μ (mu)	DQ523607	(2)
ν (nu)	DQ523615	(2)
ξ (xi)	DQ523610	(2)
ο (omicron)	AJ876648	Blanco et al., Unpublished
π (pi)	AJ705052	Blanco et al., Unpublished
ρ (rho)	DQ523613	(2)
ρ3 (rho-3)	FJ609818	Lacher et al., Unpublished
σ (sigma)	AJ781125	Blanco et al., Unpublished
τ (tau)	AY696839	(11)
υ (upsilon)	FM872417	(10)

Table S3. Reference sequences for *bfpA* allele typing (human-associated and bat-associated).

bfpA alleles	GenBank accession No.	Reference
α1 (alpha-1)	AF304474, AF304480	(13)
α2 (alpha-2)	AF304469, AF304473, AF304477, AF304485	(13)
a3 (alpha-3)	AF304468, AF304470, AF304472, AF304484	(13)
a5 (alpha-5)	FN391182	Blanco et al., Unpublished
ß1 (beta-1)	AF304471, OM925979	(13, 14)
ß2 (beta-2)	AF304486	(13)
ß3 (beta-3)	AF304476	(13)
ß4 (beta-4)	AF304478	(13)
ß5 (beta-5)	AF304475, AF304479, AF304482	(13)
ß6 (beta-6)	AF474407	(15)
ß7.1 (beta-7.1)	EF011024	(16)
ß7.2 (beta-7.2)	EF011025, EF011026	(16)
ß8 (beta-8)	FN391180	Blanco et al., Unpublished
ß10 (beta-10)	FN391178	Blanco et al., Unpublished
ß11 (beta-11)	FN391179	Blanco et al., Unpublished
ß12 (beta-12)	AB247934	(17)
ß13 (beta-13)	AB247926	(17)
ß14 (beta-14)	OM925980, AB247933	(14, 17)
FF-1.1	OM925981 and OM925982	(14)
FF-2.1	OM925983	(14)
FF-3.1	OM925984	(14)
FF-4.1	OM925985 and OM925986	(14)
FF-5.1	OM925987	(14)

References for supplementary tables

1. Elliott SJ, Wainwright LA, McDaniel TK, Jarvis KG, Deng Y, Lai LC, et al. The complete sequence of the locus of enterocyte effacement (LEE) from enteropathogenic *Escherichia coli* E2348/69. *Mol Microbiol*. 1998;28(1):1-4.
2. Lacher DW, Steinsland H, Whittam TS. Allelic subtyping of the intimin locus (*eae*) of pathogenic *Escherichia coli* by fluorescent RFLP. *FEMS Microbiol Lett*. 2006;261(1):80-7.
3. Hayashi T, Makino K, Ohnishi M, Kurokawa K, Ishii K, Yokoyama K, et al. Complete genome sequence of enterohemorrhagic *Eschelichia coli* O157: H7 and genomic comparison with a laboratory strain K-12. *DNA Res*. 2001;8(1):11-22.
4. Voss E, Paton AW, Manning PA, Paton JC. Molecular Analysis of Shiga Toxigenic *Escherichia coli* O111: H− Proteins Which React with Sera from Patients with Hemolytic-Uremic Syndrome. *Infect Immun*. 1998;66(4):1467-72.
5. Tarr CL, Large TM, Moeller CL, Lacher DW, Tarr PI, Acheson DW, et al. Molecular characterization of a serotype O121: H19 clone, a distinct Shiga toxin-producing clone of pathogenic *Escherichia coli*. *Infect Immun*. 2002;70(12):6853-9.
6. Jores J, Zehmke K, Eichberg J, Rumer L, Wieler LH. Description of a novel intimin variant (type ζ) in the bovine O84: NM verotoxin-producing *Escherichia coli* strain 537/89 and the diagnostic value of intimin typing. *Exp Biol Med*. 2003;228(4):370-6.
7. Tarr CL, Whittam TS. Molecular evolution of the intimin gene in O111 clones of pathogenic *Escherichia coli*. *J Bacteriol*. 2002;184(2):479-87.
8. Arbeloa A, Blanco M, Moreira FC, Bulgin R, Lopez C, Dahbi G, et al. Distribution of *espM* and *espT* among enteropathogenic and enterohaemorrhagic *Escherichia coli*. *J Med Microbiol*. 2009;58(Pt 8):988.
9. Blanco M, Schumacher S, Tasara T, Zweifel C, Blanco JE, Dahbi G, et al. Serotypes, intimin variants and other virulence factors of *eae* positive *Escherichia coli* strains isolated from healthy cattle in Switzerland. Identification of a new intimin variant gene (*eae*-η2). *BMC Microbiol*. 2005;5(1):23.
10. Yamamoto D, Hernandes RT, Blanco M, Greune L, Schmidt MA, Carneiro SM, et al. Invasiveness as a putative additional virulence mechanism of some atypical Enteropathogenic *Escherichia coli* strains with different uncommon intimin types. *BMC Microbiol*. 2009;9(1):1-10.
11. Hyma KE, Lacher DW, Nelson AM, Bumbaugh AC, Janda JM, Strockbine NA, et al. Evolutionary genetics of a new pathogenic *Escherichia* species: *Escherichia albertii* and related *Shigella boydii* strains. *J Bacteriol*. 2005;187(2):619-28.
12. Zhang W, Köhler B, Oswald E, Beutin L, Karch H, Morabito S, et al. Genetic diversity of intimin genes of attaching and effacing *Escherichia coli* strains. *J Clin Microbiol*. 2002;40(12):4486-92.
13. Blank TE, Zhong H, Bell AL, Whittam TS, Donnenberg MS. Molecular variation among type IV pilin (*bfpA*) genes from diverse enteropathogenic *Escherichia coli* strains. *Infect Immun*. 2000;68(12):7028-38.
14. McDougall F, Gordon D, Robins-Browne R, Bennett-Wood V, Boardman WS, Graham PL, Power M. Characterisation of typical enteropathogenic *Escherichia coli* (tEPEC) lineages and novel *bfpA* variants detected in Australian fruit bats (*Pteropus poliocephalus*). *Sci Total Environ*. 2023;902:166336.
15. Blank TE, Lacher DW, Scaletsky IC, Zhong H, Whittam TS, Donnenberg MS. Enteropathogenic *Escherichia coli* O157 strains from Brazil. *Emerg Infect Dis*. 2003;9(1):113.
16. Lacher DW, Steinsland H, Blank TE, Donnenberg MS, Whittam TS. Molecular evolution of typical enteropathogenic *Escherichia coli*: clonal analysis by multilocus sequence typing and virulence gene allelic profiling. *J Bacteriol*. 2007;189(2):342-50.
17. Iida M, Yamazaki M, Yatsuyanagi J, Ratchtrachenchai OA, Subpasu S, Okamura N, Ito K. Typing of *bfpA* genes of enteropathogenic *Escherichia coli* isolated in Thailand and Japan by heteroduplex mobility assay. *Microbiol immunol*. 2006;50(9):713-7.
